# Immunophenotyping of bronchoalveolar lavage and functional impairment in post-COVID syndrome: Insights from a prospective cohort trial

**DOI:** 10.1007/s00508-025-02531-9

**Published:** 2025-04-22

**Authors:** Maximilian Robert Gysan, Antje Lehmann, Dominik Bernitzky, Andreas Zech, Jonas Brugger, Helmut Prosch, Marco Idzko, Daniela Gompelmann

**Affiliations:** 1 Division of Pulmonology, Department of Internal Medicine II, Medical University of Vienna, Währinger Gürtel 18–20, 1090 Vienna, Austria; 2 Institute for Medical Statistics, Medical University of Vienna, Vienna, Austria; 3 Department of Biomedical Imaging and Image-guided Therapy, Medical University of Vienna, Vienna, Austria; 4 Department of Thoracic Surgery, Medical University of Vienna, Vienna, Austria

**Keywords:** COVID-19 pneumonia, Immunopathology, Lung function, SARS-CoV‑2, Inflammation

## Abstract

**Objective:**

Following recovery from COVID-19, there is evidence for pulmonary sequelae and functional impairment. Data regarding the immunopathological mechanisms are limited. This study aimed to investigate the relationship between bronchoalveolar lavage fluid (BALF) cellularity, lung function impairment and high-resolution computed tomography (HRCT) changes in post-COVID syndrome patients.

**Methods:**

Patients with post-COVID syndrome were enrolled in this Austrian single-center prospective observational cohort study. All patients underwent a pulmonary function test (PFT) and chest HRCT. Those with pathological HRCT findings underwent bronchoscopy with BALF sampling for differential cell count and fluorescence-activated cell sorting analysis.

**Results:**

In this study 26 patients with post-COVID syndrome underwent bronchoscopy with BAL. The HRCT showed ground-glass opacifications (69.2%), organizing pneumonia (7.7%) or both (11.5%). The PFT revealed restrictive lung disease in 38.5% and reduced diffusion capacity in 68%, 19.2% showed a pathological BAL cell pattern predominantly consisting of CD4^+^ T‑cells. The BALF lymphocyte count was associated with reduced forced vital capacity (*p* = 0.016) and an elevated alveolar-arterial oxygen gradient (*p* = 0.04).

**Conclusion:**

A notable percentage of patients with post-COVID syndrome with persistent HRCT changes showed T‑helper lymphocytic inflammation in the lungs. The degree of alveolar lymphocytosis was associated with lung function impairment. This could suggest that a prolonged inflammatory response in the alveolar compartment contributes to the pathogenesis of post-COVID syndrome.

## Introduction

Severe acute respiratory syndrome coronavirus 2 (SARS-CoV-2) is the pathogen causing the coronavirus disease 2019 (COVID-19) pandemic with over 777 million cases by 8 December 2024 [1]. Following the acute viral infection, a post-acute viral syndrome emerged similar to the previous coronavirus pandemics caused by SARS and Middle East Respiratory Syndrome (MERS) affecting various organ systems [2, 3]. Symptoms include dyspnea, cough, fatigue, cognitive impairment, anxiety, depression, chest pain and arthralgia [4–6]. Persistent symptoms beyond 4 weeks from the onset of an acute COVID-19 infection are referred to as post-acute COVID or post-acute sequelae of COVID-19 [7]. Persistent symptoms for more than 12 weeks are referred to as post-COVID syndrome [7, 8]. Pathophysiological mechanisms can include persistence of viruses or viral components leading to tissue damage, chronic hyperinflammation, autoimmune phenomena and a dysregulated fibrinolytic system [9, 10].

Data on the prevalence of persistent symptoms following COVID-19 vary greatly due to differences in the study design, patient populations, observation period and the assessment of symptoms (e.g., telephone survey vs. inpatient visits). In a review of 120 studies assessing the prevalence of post-COVID syndrome, a pooled estimate of 34.5% of patients reported persisting symptoms more than 12 weeks following COVID-19 infection [11]. Given the incidence of COVID-19, this affects a substantial number of patients.

Restrictive lung disease, reduced diffusion capacity, changes on high-resolution computed tomography (HRCT) including ground-glass opacifications, irregular lines, consolidations and reticulations have been reported at follow-up of COVID-19 patients [6, 12]. Bronchoscopy with bronchoalveolar lavage is a well-established test in the evaluation of interstitial lung disease to obtain diagnostic and prognostic information [13]. Most studies investigating bronchoalveolar lavage fluid (BALF) in COVID-19 patients focused on critically ill patients with acute respiratory distress syndrome, who developed neutrophilic alveolitis [14–16]; however, only few studies have investigated BALF for cellular analyses in patients with post-COVID syndrome. At this time, there are no studies linking BALF cell analysis to lung functional impairment or structural changes on imaging related to COVID-19. In this study, we investigated to what extent changes in BALF cellularity occur in patients with post-COVID syndrome and persistent changes on HRCT and whether there is an association between BALF cellularity and lung functional impairment.

## Patients, material and methods

### Study design

This prospective observational cohort study was conducted at the Department of Pulmonology at the Medical University of Vienna. Patients who experienced COVID-19 in the past 6 months and were referred to the post-COVID outpatient clinic for medical assessment independent of symptoms or severity of COVID-19 were evaluated for participation. This study was performed in accordance with the Declaration of Helsinki and was approved by the ethics committee of the Medical University of Vienna (1551/2020). All adult participants provided written informed consent to participate in this study. Inclusion criteria for study enrolment were a PCR-confirmed diagnosis of COVID-19 in adults aged ≥ 18 years with persistent symptoms. Exclusion criteria were cognitive impairment, contraindications for performing bronchoscopy with BALF sampling (e.g., respiratory limitations with DLCO < 25% or FEV1 < 800 mL), pre-existing concomitant pulmonary disease (such as cystic fibrosis, alpha-1-antitrypsin deficiency, chronic obstructive pulmonary disease, interstitial lung disease, asthma or sarcoidosis), malignancy, severe cardiovascular disease and a history of unstable angina or a heart attack 1 month prior to study inclusion. The following examinations were included in the screening process: medical history including patients demographics, onset and nature of symptoms and comorbidities, pulmonary function test (PFT) including total lung capacity (TLC), forced vital capacity (FVC), forced expiratory volume in 1 s (FEV1), as well as blood gas analysis, diffusion capacity of the lung for carbon monoxide (DLCO), carbon monoxide transfer coefficient (DLCO/VA), blood tests, high-resolution computed tomography (HRCT) and cardiopulmonary exercise test (CPET). The HRCT findings were evaluated by a radiologist in multidisciplinary meetings for interstitial lung disease. Patients with persistent changes on HRCT underwent a bronchoscopy with BALF sampling. The severity of COVID-19 during the acute phase was assessed using the World Health Organization (WHO) clinical progression scale [17].

**Fig. 1:**
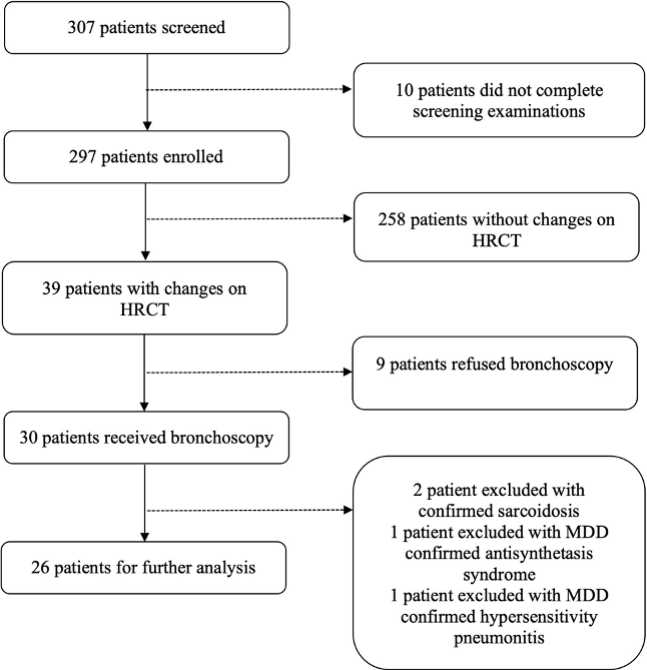
Flow chart of the study population. *MDD* Multidisciplinary discussion

The enrolment process is illustrated in Fig. 1. Patients were enrolled from May 2020 until July 2022. In this sub-analysis, we reviewed patients who received bronchoscopy with BALF sampling. Out of 297 patients who were enrolled, 30 received a bronchoscopy with BALF sampling and 4 patients were excluded from further analysis due to a concomitant pulmonary disease that was diagnosed during the course of the study. In the remaining 26 patients SARS-CoV‑2 infection was confirmed by PCR between 19 March 2020 and 3 April 2021 and patients were enrolled between 4 June 2020 and 31 August 2021. Thus, the patients were infected with either the SARS-CoV‑2 wild-type, alpha variant, beta variant or gamma variant.

### Assessment of BALF

The bronchoscope was positioned in the segmental or subsegmental bronchus of the middle lobe or lingula. Up to 300 ml of sterile saline was gradually injected and withdrawn in steps using a handheld syringe. The BALF was collected and analyzed for total cell count. For patients with ≥ 11% lymphocytes in the BALF samples, flow cytometry was conducted to assess the CD4/CD8 ratio and the proportions of CD3^+^ T cells, CD4^+^ helper T cells, CD8^+^ cytotoxic T cells, B cells (CD19^+^), and senescent T cells (CD57^+^). After counting and washing, Fc receptors were blocked for 30 min with Human TruStain FcX (BioLegend Inc., San Diego, CA, USA), followed by a 20-min fluorescence-activated cell sorting (FACS) staining process using phosphate-buffered saline with 0.5% bovine serum albumin and 0.01% sodium azide. Differential cell counts were evaluated using flow cytometry. The analysis was conducted on a CytoFlex LS (Beckman Coulter Inc., Pasadena, CA, USA) using Cellquest version 3.3 (BD Biosciences Inc., Franklin Lakes, NJ, USA) and FlowJo version 6.4.7 (Tree Star Inc., San Carlos, CA, USA) software. Thresholds for BAL cell patterns were based on the Americal Thoracic Society clinical practice guidelines, with cut-offs set at > 15% for lymphocytes, > 3% for neutrophils and > 1% for eosinophils [18].

### Statistical analysis

The BALF lymphocyte counts were correlated with pathological findings in PFTs and serological markers using Spearman’s rank correlation coefficients for metric variables and Mann-Whitney U‑tests for dichotomized parameters. To assess the correlation of each explanatory parameter with the lymphocyte count, Spearman’s Rho and the corresponding significance level were computed for each parameter separately. With the dichotomized parameters, group comparisons were performed by computing Mann-Whitney U‑tests with lymphocyte count as the target variable. Group comparisons were also performed with respect to the occurrence of autoimmune features. No correction for multiple testing was applied, therefore all *p*-values are of a descriptive, hypothesis-generating character. Statistical analysis was done using SPSS version 29 (SPSS Inc., Chicago, IL, USA).

## Results

In total 297 patients were enrolled in this prospective trial out of which 26 patients underwent bronchoscopy with BALF for COVID-19-associated changes on HRCT. The median age of these 26 patients was 57 years (range 32–78 years). Bronchoscopy was performed after a median of 105 days (interquartile range, IQR 90–155 days) following initial diagnosis of COVID-19. Leading symptoms included dyspnea (61.5%), fatigue (19.2%), cough (15.4%) and chest pain (11.5%).

Overall, 61.5% of patients (*n* = 16) were initially hospitalized for acute COVID-19 pneumonia, 42.3% of patients (*n* = 11) did not require respiratory support, 23.1% (*n* = 6) received oxygen therapy, 19.2% (*n* = 5) received noninvasive ventilation or high-flow nasal oxygen therapy, 4 patient required mechanical ventilation out of which 3 patients required extracorporeal membrane oxygenation. Out of the 16 patients hospitalized for acute COVID-19 pneumonia, 26.9% (*n* = 7) received therapy with dexamethasone, 2 patients received hydroxychloroquine, 2 patients received tocilizumab and 2 patients received the serine protease inhibitor camostat. Of the patients 15.4% received antiviral therapy, i.e., a combination of lopinavir and ritonavir (*n* = 2), remdesivir (*n* = 1) and favipiravir (*n* = 1). Antithrombotic therapy was administered in 42.3% of patients (*n* = 11), 34.6% of patients (*n* = 9) received antibiotic treatment for acute COVID-19 pneumonia due to secondary bacterial infections. All of these patients were hospitalized and presented with moderate to severe COVID-19 pneumonia, i.e., a WHO progression scale ≥ 5.

Patients’ demographics are shown in Table 1. The most frequently occurring comorbidities were arterial hypertension and diabetes. Cardiac disease included atrial fibrillation (*n* = 1) and valvular heart disease (*n* = 1). Vascular disease included one patient each with peripheral artery occlusive disease, deep vein thrombosis, pulmonary embolism and chronic venous insufficiency.

**Table 1:** Patient demographics

Patients (*n*)	26
*Female:male (n)*	5:21
*Age in years (median, range)*	57 (32–78)
*Hospitalization for acute COVID-19 (n, %)*	16 (61.5)
WHO clinical progression scale (median, IQR)	5 (3–6)
Length of hospital stay in days (median, IQR)	24 (10–46.5)
**Smoking status**	
Never smokers (*n*, %)	11 (42.3)
Past smokers (*n*, %)	13 (50)
Current smokers (*n*, %)	2 (7.7)
*BMI (median, IQR)*	29 (28–32)
**Comorbidities**	
Arterial hypertension (*n*, %)	9 (34.6)
Cardiac disease (*n*, %)	2 (7.7)
Cancer (*n*, %)	2 (7.7)
Diabetes (*n*, %)	7 (26.9)
Chronic kidney disease (*n*, %)	1 (3.8)
Vascular (*n*, %)	4 (15.4)
**Pulmonary function test**	
TLC in % predicted (mean, SD)	89.7 ± 18.5
TLC in ml (mean, SD)	5919 ± 1321
FVC in % predicted (mean, SD)	84.3 ± 20.7
FVC in ml (mean, SD)	3670 ± 1041
FEV1 in % predicted (mean, SD)	85.8 ± 19.6
FEV1 in ml predicted (mean, SD)	2924 ± 857
FEV1/FVC in % predicted (mean, SD)	79.5 ± 6.9
DLCO in % predicted (mean, SD)	68.5 ± 19.4
DLCO/VA in % predicted (mean, SD)	87.9 ± 16.2
AaDO_2_ in mm Hg (mean, SD)	17.1 ± 9
*Days between initial diagnosis of COVID-19 and bronchoscopy with BALF sampling (medians, IQR)*	103 (90–155)

*AaDO*_*2*_ alveolar-arterial oxygen gradient, *BMI* body mass index, *DLCO* diffusing capacity of the lungs for carbon monoxide, *DLCO/VA* CO transfer coefficient, *FEV*1 forced expiratory volume in 1 s, *FVC* forced vital capacity, *IQR* interquartile range, *SD* standard deviation, *TLC* total lung capacity

### HRCT, PFT and laboratory findings

All patients showed pathological findings on HRCT, i.e., ground-glass opacifications (69.2%, *n* = 18), HRCT findings compatible with organizing pneumonia (7.7%, *n* = 2), ground-glass opacifications and HRCT findings compatible with organizing pneumonia (11.5%, *n* = 3), nonspecific nodules and bronchiectasis in 1 patient each. The PFT revealed a restrictive lung disease defined as TLC below the lower limit of normal in 38.5% (*n* = 10) and a reduced DLCO < 80% predicted in 68% (*n* = 17). In 27% (*n* = 7) of patients ANA-titres ≥ 1:80, specific ENAs (anti-smooth muscle antibodies, anti-SSA/Ro antibodies), rheumatoid factor or cyclic citrullinated peptide antibodies were identified, which we defined as autoimmune features. Patients who were hospitalized for COVID-19 during the acute phase presented with lower FVC than ambulatory patients (*p* = 0.027). Patients hospitalized with severe disease defined as WHO progression scale ≥ 6 presented with lower FVC than patients with WHO progression scale < 6 (*p* = 0.004) (Table 2).

**Table 2:** Association of severity of COVID-19 with forced vital capacity

Variable	FVC in % predicted (mean, SD)		*p*-value
WHO clinical progression scale ≥ 4 vs. < 4	77.1 ± 18.0	96.4 ± 19.8	0.027
WHO clinical progression scale ≥ 6 vs. < 6	68.7 ± 16.9	92.8 ± 17.6	0.004

*FVC* forced vital capacity, *SD* standard deviation

### BALF results

**Fig. 2:**
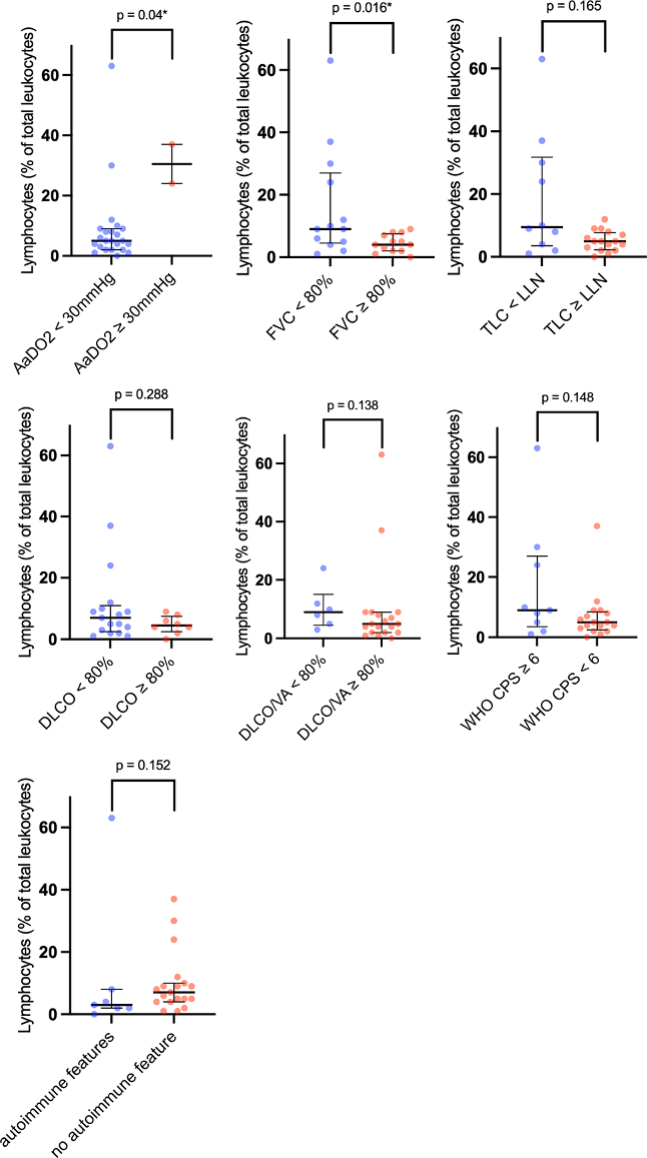
BALF lymphocyte counts by clinical and pulmonary function variables. Data are shown as scatter plots (median with interquartile range) with *each dot* representing an individual patient sample (*p*-values are shown above brackets). *FVC* forced vital capacity, *LLN* lower limit of normal, *TLC* total lung capacity, *DLCO* diffusions capacity of the lung for carbon monoxide, *DLCO/VA* carbon monoxide transfer coefficient, *AaDO2* alveolar-arterial oxygen gradient, *WHO CPS* World Health Organization clinical progression scale

Of the patients 42.3% (*n* = 11) showed elevated BAL cell count, defined as > 100 cells/µl (see Table 3). A lymphocytic BAL cell pattern was found in two patients, a mixed pattern with elevated lymphocytes and neutrophils or eosinophils in two patients and an eosinophilic pattern in one patient. The BALF lymphocyte phenotyping via FACS was available in four patients revealing that CD4^+^ cells were the predominant lymphocytes. The FVC correlated significantly with the BALF lymphocyte count with a Spearman’s Rho of −0.407 (*p* = 0.039) (Table 4; Fig. 2). Also, patients with an AaDO_2_ ≥ 30 mm Hg showed a significantly higher lymphocyte count of 30.5 compared to patients with an AaDO_2_ < 30 mm Hg (Table 5); however, the comparison is skewed, with only 2 patients having an AaDO_2_ ≥ 30 mm Hg, while the remaining 24 patients had an AaDO_2_ < 30 mm Hg, as shown in Fig. 2. Of the patients three received prednisolone at the time of bronchoscopy in a dosage between 2.5 mg and 5 mg daily and one patient received prednisolone due to a kidney transplantation. The association of clinical and pulmonary function variables with the BALF lymphocyte count is displayed in Tables 4 and 5.

**Table 3:** BALF metrics, differential cell counts and FACS

Variable	Value
*Volume of BALF recovery in ml (median, IQR)*	140 (120–180)
*BALF total cell count in cells/μL (median, IQR)*	81 (50–150)
*BALF differential cell counts*	
Macrophages in % (median, IQR)	92 (88–96)
Neutrophils in % (median, IQR)	2 (2–3)
Lymphocytes in % (median, IQR)	5.5 (3–9)
Eosinophils in % (median, IQR)	3 (1–6)
*CD3 in % (median, IQR)*	77.7 (73–83)
*CD4 in % (median, IQR)*	48.2 (34–62)
*CD8 in % (median, IQR)*	26.2 (18–34)
*CD4/CD8 ratio (median, IQR)*	3.8 (2–6)
*CD56 in % (median, IQR)*	6.3 (4–8)

*BALF* bronchoalveolar lavage fluid; *IQR*, interquartile range

**Table 4:** Correlation of clinical and pulmonary function metrics with BALF lymphocyte count

Variable	Spearman’s Rho	*p*-value
AaDO2	0.169	0.418
FVC % predicted	−0.407	0.039
TLC % predicted	−0.310	0.123
DLCO % predicted	−0.206	0.324
DLCO/VA % predicted	−0.016	0.941
WHO clinical progression scale	0.164	0.425
CRP at discharge (*n* = 16)	−0.074	0.785
Length of hospital stay	0.131	0.628
Smoking status	−0.078	0.705
Immunomodulatory or antiviral therapy for acute COVID-19	0.154	0.452

*AaDO2* alveolar-arterial oxygen gradient, *CRP* C‑reactive protein, *DLCO* diffusing capacity of the lungs for carbon monoxide, *DLCO/VA* CO transfer coefficient, *FVC* forced vital capacity, *TLC* total lung capacity

**Table 5:** Association of dichotomous clinical and pulmonary function variables with BALF lymphocyte count

Variable	BALF lymphocytes in % (median, IQR)		*p*-value
AaDO2 ≥ 30 mm Hg vs. < 30 mm Hg	30.5 (24–37)	5 (2–9)	0.040
FVC ≥ 80% predicted vs. < 80%	9 (4.5–27)	4 (2–7.5)	0.016
TLC ≥ 80% predicted vs. < 80%	5 (2.5–8)	9.5 (2.75–31.75)	0.165
DLCO ≥ 80% predicted vs. < 80%	4.5 (2.5–7.5)	7 (2.5–11)	0.288
DLCO/VA ≥ 80% predicted vs < 80%	5 (2–9)	9 (4.5–15)	0.138
Autoimmune features yes vs. no	3 (2–8)	7 (4–10)	0.152
WHO clinical progression scale ≥ 4 vs. < 4	6.5 (2.25–11.5)	5 (3.5–9)	0.698
WHO clinical progression scale ≥ 6 vs. < 6	9 (3.5–27)	5 (2.5–8.5)	0.148

*AaDO2* alveolar-arterial oxygen gradient, *DLCO* diffusing capacity of the lungs for carbon monoxide, *DLCO/VA* CO transfer coefficient, *IQR* interquartile range, *FVC* forced vital capacity, *TLC* total lung capacity

## Discussion

In this prospective, monocentric observational study, patients with post-COVID syndrome and ground-glass opacifications or organizing pneumonia on chest imaging underwent bronchoscopy with BAL. Restrictive lung disease was observed in 38.5% of patients, while 68% exhibited a reduced diffusion capacity. A lymphocytic BAL cell pattern was identified in approximately 20% of patients. The T‑helper lymphocytic inflammation in the lungs was associated with reduced forced vital capacity and an elevated alveolar-arterial oxygen gradient.

The clinical presentation of our patients with post-COVID syndrome was similar to those observed in previous cohorts: 61.5% of patients presented with dyspnea, 19.2% of patients reported persistent fatigue, cough was reported by 15.4% and chest pain by 11.5% of patients.

In a study of patients with post-acute COVID, Townsend et al. reported that more than half presented with persistent fatigue 10 weeks after initial COVID-19 infection [19]. Another trial on persistent symptoms in a post-acute COVID cohort showed that 43.5% of patients reported dyspnea 60 days after the onset of the first COVID-19 symptoms [20]. In comparison, in a large prospective cohort study including 12,947 participants with post-COVID syndrome 22.2% of patients experienced dyspnea, 46.8% reported tiredness, 22.9% had either dry cough or cough with phlegm and 7.1% experienced chest pain 6 months after symptomatic SARS-CoV‑2 infection [21]. Bowe et al. could demonstrate that even 2 years after the initial infection with SARS-CoV‑2, the risk for pulmonary sequelae and fatigue remained increased compared to a control group without a history of SARS-CoV‑2 infection [22].

Compared to the findings of Carfi et al. our cohort exhibited a higher prevalence of dyspnea. This discrepancy might be attributed to the fact that our study specifically includes patients with persistent CT abnormalities, potentially representing a subgroup with more severe manifestations.

Here, we could demonstrate that in a cohort of 297 patients with outpatient or hospitalized COVID-19 disease, around 13.1% (*n* = 39) showed CT changes after a median of 77 days with ground-glass opacifications being the predominant HRCT pattern. In line with this study, Huang et al. could demonstrate that ground-glass opacifications were the predominant HRCT pattern 6 months after symptom onset for COVID-19 ranging from 41% in patients with no supplemental oxygen therapy to 45% in patients requiring high-flow nasal canula non-invasive ventilation or mechanical ventilation for initial SARS-CoV‑2 pneumonia [6]. In contrast to this study, only 2% of patients presented with perilobular opacifications or consolidations suggestive of organizing pneumonia in the study of Huang et al. [6]. Han et al. found fibrotic-like changes in 35% and residual ground-glass opacifications in 27% of patients 175 days after the onset of symptoms for COVID-19 [23]. The relatively high proportion of fibrotic-like changes in HRCT might be explained by the fact that 63% of these patients had developed severe SARS-CoV‑2 infection with acute respiratory distress syndrome (ARDS).

Huang et al. found that impairment in pulmonary function tests 6 months after symptom onset depends on the severity of the initial COVID-19 infection [6]. Only 11% of patients who did not require oxygen therapy for an initial COVID-19 infection developed restrictive lung disease, defined as TLC < 80% of the predicted value, whereas the proportion was 30% among patients who required ventilation. A similar effect was observed for diffusion capacity with 56% of patients developing lung diffusion impairment defined as DLCO < 80% predicted following ventilation therapy compared to merely 22% in patients who required no oxygen therapy [6]. Similarly, in this study FVC was significantly lower in patients with severe COVID-19 during the acute phase defined by the WHO clinical progression scale. Wu et al. report a reduced diffusion capacity defined as DLCO < 80% in 55% of patients and FVC < 80% in 23% after 3 months in a cohort of 83 patients who recovered from severe COVID-19 [24]. Another study including 54 patients found lung diffusion impairment defined as DLCO < 80% in 32.1% after a follow-up period of 6 months [25]. In this study we demonstrate that 38.5% (*n* = 10) of patients had a restrictive lung disease and 68% (*n* = 17) presented with a reduced diffusion capacity. The comparatively high proportion of patients with manifest restrictive lung disease and reduced diffusion capacity could result from the selection bias including only patients with persistent HRCT changes in the analysis; however, in this study all patients with pre-existing lung disease were excluded from the analysis unlike the work of Huang et al. [6].

In this study post-COVID syndrome patients with persistent changes on HRCT were assessed with BALF sampling and 11 out of 26 patients showed an elevated cell count with lymphocytosis being the predominant pattern. The FVC correlated with alveolar lymphocyte count in BALF. Furthermore, AaDO2 ≥ 30 mm Hg in blood gas was associated with a higher lymphocyte count; however, prolonged lymphocytic inflammation in BALF was not associated with the severity of COVID-19 during the acute phase or post-COVID inflammation, defined as the C‑reactive protein (CRP) value at hospital discharge. Previous studies demonstrated that in patients with acute COVID-19 pneumonia receiving intensive care unit (ICU) treatment, neutrophils were the predominant leucocyte in BALF sampling and associated with poor survival [14–16]; however, data on BALF in post-COVID syndrome are scarce. Steinestel et al. were able to detect a mean lymphocyte count of 12.5% in BALF in 33 patients with mild to moderate COVID-19 and persistent respiratory symptoms [26]. Rolland-Debord et al. performed bronchoscopy with BALF sampling in patients with respiratory symptoms or radiological abnormalities 36–109 days after a documented COVID-19 pneumonia [27]. The authors could show an elevated cell count in the BALF and 62% of patients showed CD4^+^ dominant lymphocytosis, defined as > 10% of cells. Similarly, CD4^+^ T‑cells were the predominant lymphocytes in BALF sampling in this study; however, BALF lymphocyte phenotyping via FACS was only performed in 4 patients. We could identify a significant BALF lymphocytosis in 15.4% of patients suggestive of alveolar inflammation and an association between BALF lymphocyte count and changes in PFT.

It can be hypothesized that a prolonged inflammatory response damages local tissue structures by disrupting immune homeostasis, leading to functional impairments [28]. This may indicate a pathogenic link in post-COVID syndrome and suggest an association between BALF lymphocyte count and disease activity.

Berentschot et al. demonstrated that T‑lymphocyte counts and late stage differentiated CD8^+^ T‑cell counts in blood samples were increased in patients with post-COVID syndrome compared to healthy controls [29]. Other studies have shown an association between elevated total lymphocytes, NK-cells, CD8^+^ T cell counts and the persistence of symptoms in patients with post-COVID syndrome compared to healthy controls or patients with symptoms of COVID-19 that were completely resolved [30, 31].

We excluded 4 patients from further analysis, who were diagnosed with pulmonary disease during the course of the study, i.e. sarcoidosis (*n* = 2), hypersensitivity pneumonitis (*n* = 1) and interstitial lung disease due to antisynthetase syndrome (*n* = 1). Kim et al. could demonstrate in a large population-based cohort study that the risk of developing interstitial lung disease was elevated in a cohort of patients who had SARS-CoV‑2 infections compared to a control group [32]. Another study found that patients with a history of COVID-19 had a higher risk of developing new-onset asthma or bronchiectasis compared to individuals without COVID-19 [33].

This study provides original findings in patients with post-COVID syndrome, nevertheless there are limitations. The number of patients with post-COVID syndrome who underwent bronchoscopy with BALF sampling was limited, especially the number of those who underwent FACS for leucocyte phenotyping. A further limitation is that only patients with post-COVID syndrome and concomitant CT changes were examined.

In conclusion we could demonstrate that a relevant percentage of patients with post-COVID syndrome develop CD4^+^ dominant lymphocytic alveolitis. The degree of alveolar lymphocytosis was associated with impairment in pulmonary function tests and might therefore indicate a prolonged inflammatory response of the alveolar compartment as a pathogenic mechanism for post-COVID syndrome.

## Conclusion

Post-COVID syndrome affects a substantial number of patients. In patients with post-COVID syndrome and persistent changes on HRCT we could identified a link between alveolar lymphocytosis, particularly CD4^+^ dominant lymphocytic alveolitis and pulmonary function impairment. Alveolar lymphocytosis signifies a prolonged inflammatory response of the alveolar compartment as a potential pathogenic mechanism for post-COVID syndrome.

## Data Availability

The datasets used in the current study are available from the corresponding author on reasonable request.

## Abbreviations

AaDO_2_: Alveolar-arterial oxygen gradient
ANA: Antinuclear antibody
Anti-SSA/Ro antibodies: Anti–Sjögren’s-syndrome-related antigen A antibodies
BAL: Bronchoalveolar lavage
BALF: Bronchoalveolar lavage fluid
DLCO: Diffusing capacity of the lungs for carbon monoxide
DLCO/VA: Carbon monoxide transfer coefficient
ENA: Extractable nuclear antigen
FACS: Fluorescence-activated cell sorting
FEV1: Forced expiratory volume in 1 s
FVC: Forced vital capacity
HRCT: High-resolution computed tomography
MDD: Multidisciplinary discussion
PCR: Polymerase chain reaction
PFT: Pulmonary function test
SAA: Serum amyloid A
TLC: Total lung capacity
